# Addressing the Challenging Problem of High-Riding NAC after Breast Reduction: A Novel Solution and Review of Techniques

**DOI:** 10.1055/s-0045-1804926

**Published:** 2025-03-05

**Authors:** Rajat Gupta, Priya Bansal, Neharika Neeraj

**Affiliations:** 1 Department of Aesthetic and Plastic Surgery, C K Birla Hospital, New Delhi, India

**Keywords:** breast reduction, high-riding nipple, two-flap technique, mastopexy

## Abstract

High-riding nipple–areolar complex (NAC) due to postoperative malposition following breast reduction surgery is a very serious aesthetic problem for the patients and a very difficult one for surgeons to correct. Reduction surgeries aim to elevate the NAC, and the best course of action for avoiding a high-riding NAC is prevention of over-elevation, taking care of marking the appropriate distance between the NAC and the inferior mammary fold. Its correction poses a very difficult challenge due to the limited skin between the upper edge of the NAC and the sternal notch and the concern for avoiding scars that lie above the nipple in the superior pole of the breast. There are various techniques described for repositioning the NAC to an acceptable position, but most of them come with the drawback of unsatisfactory correction of bottoming out, additional scars, and multiple stages. A technique of mastopexy called “two-flap technique” including repositioning of the NAC as well as elevation of inferior breast mound (or correction of bottoming out), without any additional scars, is described in this article.

## Introduction

One of the complications of breast reduction surgeries is a high-riding nipple–areolar complex (NAC). 1 While one of the goals of breast reduction surgery is to elevate the NAC, it sometimes gets elevated more than ideal. Its presentation can vary from the NAC being located slightly above the point of maximum projection to a very visibly misplaced high-riding NAC that appears very unaesthetic and is disturbing for the patient. This problem can be compounded by a bottoming out of breast tissue over the next few months and then the overriding NAC looks much higher.

**Fig. 1:**
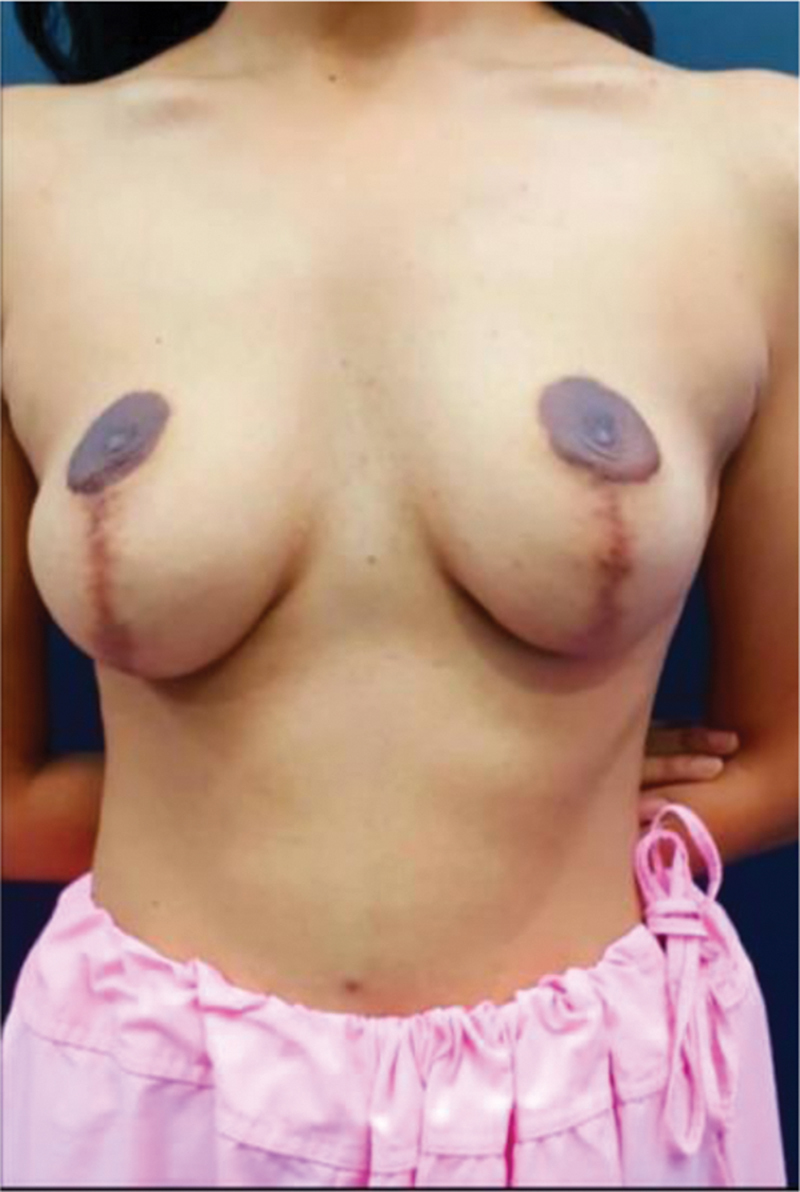
Preoperative photo.

Preoperatively the site of the NAC is marked such that it sits on the area of maximum breast projection irrespective of the tissue pedicle design or skin excision pattern chosen. Postoperative NAC displacement has been identified in long-term follow-up by various authors and this is why some advocate marking the new NAC position 1.5 to 1.75 cm below the most projected area intraoperatively ( Fig. 1 ). 2 3 4

The importance of achieving a suitable NAC position following reduction mammoplasty is paramount due to both the risk of development of a high-riding NAC and the difficult problem it poses in terms of its management. The management of this complication takes into account the nature and degree of displacement of the NAC. Several surgical options have been described to correct the same, including the elevation of the inframammary fold (IMF) and the breast parenchyma, expanding the skin above the NAC, and transposing or directly repositioning the nipple by excising it and grafting it in the required location. 5 With nearly all these techniques, there are the issues of leaving unavoidable scars above the superior aspect of the NAC and the limited amount of skin available between the nipple and the clavicle. In this article, a single-stage technique of addressing this problem has been described, which is a modification of “auto-augmentation mastopexy technique” without giving any additional scars on the superior pole of breasts.

## Case Report

A 29-year-old lady presented to the author after 4 months of breast reduction surgery with the complaint of an abnormally situated NAC. The details of the operative procedure were not known, but on examination, a scar suggestive of Wise pattern skin excision was present. Other examination findings were a high-riding NAC and a large distance between the inferior edge of the NAC and the IMF.

Many times, when a breast reduction surgery is performed, a point that is missed is that an adequate distance of 6 to 8 cm should be kept between the NAC and the IMF. If this is not kept in mind, then excess skin is left behind in the inferior pole and the IMF does not get properly raised, which in turn promotes bottoming out of the breast tissue. This, along with the already slightly higher placed NAC, compounds the problem and gives the whole breast a very odd look.

In this secondary procedure case being reported, it was planned to raise the IMF. The surgical plan was designed such that we not only raise the IMF higher but also take the flap of the bottomed-out breast tissue and fix it in the superior pole above the NAC. This would in turn give it the shape of a normal breast. Although this technique will decrease the distance between the clavicle and the upper pole of the breast, it was discussed with the patient and her consent was taken. Apart from this, any other technique would have given her a scar on the upper pole of the breast.

After marking the old scar for excision, the skin was excised along the marking. Two parenchymal flaps were designed, the first based on the superior pedicle including the NAC and the second on the central pedicle including the inferior breast tissue. The inferior mound/tissue was shifted up, from below the NAC and its upper border hitched up to the level of the second intercostal space.

Ethibond sutures were used for the same, taking bites between the dermis of the derma-glandular mound and the pectoral fascia with superior soft tissue. After the flap was inset in place, the horizontal excess of skin in the lower pole was required to be excised. To begin with, in this case the distance between the NAC and the IMF was 12 cm, which was reduced to 6 cm. Now the new IMF was created by suturing the dermo-subcutaneous junction of skin with the fascia over the pectoralis major muscle underneath. Permanent Ethibond sutures were used to achieve the same. This technique helped raise the IMF along with the relocation of the inferior bottomed-out breast tissue to the superior pole, which created a mound in the upper pole and helped in relocating the NAC on to the center of the breast. All this was achieved without actually moving the NAC's position. The final scar was an inverted-T type. No additional scars were given. Postoperatively dressings were removed on day 2.

**Fig. 2:**
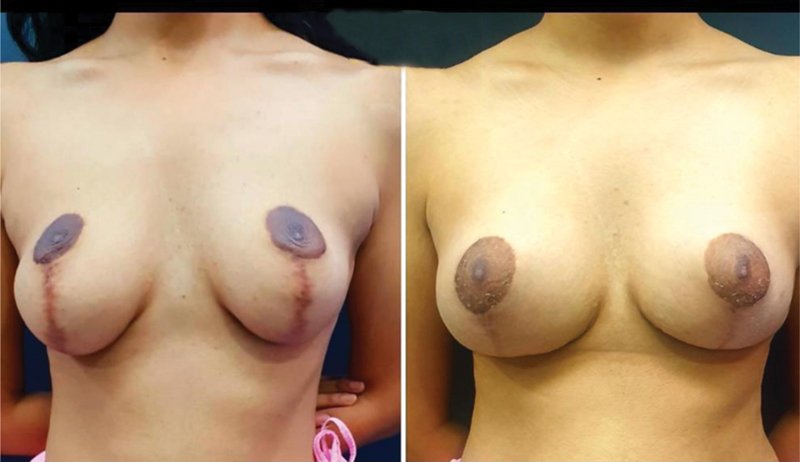
Pre-and post- operative photos.

The only limitation of this technique is the slight but inadvertent shortening of the ideal distance between the NAC and the clavicle, but practically it appears to be a good trade-off for additional scars in that area as in other techniques. Also, while a period of 6 months to allow return of softness of tissues and vascularity is advisable, in the said case, since the skin was soft, the scars settled, and the patient was in considerable distress, the surgery was performed after 4 months ( Fig. 2 ).

## Discussion



**Video 1** Auto-augmentation mastopexy video.


Breast reduction and mastopexy are commonly performed to change the size or shape of the breast, lift the breast, and raise the NAC to give the breast a more youthful appearance. But when the NAC is placed too high up, the result is unnatural and in turn the patient is unhappy. It cannot be stressed enough that it is best to avoid the creation of a high-riding nipple, which is difficult to address postoperatively. For its prevention, good preoperative evaluation of the breast while marking the new nipple position is important. Moving the position of the nipple upward is one of the desired effects of reduction mastopexy, and transposition of the IMF continues to be the most widely accepted approach for selecting a new nipple position. It is important to ensure the presence of sufficient skin between the upper breast border and the NAC as well as to avoid excess skin between the lower edge of the NAC and the IMF. 1 5 6 Increasing the breast projection helps ensure the nipple position does not displace too cranially. A superior, medial, or superomedial pedicle allows for resection of the inferior parenchyma and closure of the medial and lateral glandular flaps for improving breast projection and decreasing the risk of bottoming out. 1 5

If the nipple is elevated too high and patients are dissatisfied, there is a need for repositioning the NAC. Although various techniques have been described in the literature to correct this difficult problem, there are challenges of limited skin between the upper pole of the NAC and the sternal notch and avoiding scars superior to the NAC. Few of these techniques provide a solution to the above-mentioned two problems while achieving satisfactory elevation of the breast tissue as well as repositioning the NAC appropriately.

Location of the scars, position of the nipple with regard to its distance from the sternal notch and the IMF, position of the IMF, location of breast volume, blood supply, breast skin elasticity, and any previous radiation therapy are all considerations in selecting the most suitable surgical approach. 5 Some of the techniques address the position of the breast mound, some only the nipple position, and few address both the nipple and the breast mound.

In the classification and treatment system described by Colwell et al, lower pole remodeling is suggested for grade 1 nipple elevation (pseudo-elevation), defined as the inferior pole descent or “bottoming out,” with the nipple in a relatively fixed position. 7 In the cases where there is a near-normal nipple to sternal notch distance, a long nipple to IMF distance, a bottomed-out breast, or a depleted upper pole, inferior breast parenchyma resection with raising of the IMF may be a preferred solution ( Video 1 ). 8

Grade 2 nipple elevation has been described as nipple mildly superiorly displaced, with or without inferior pole descent. For grade 2 elevation, a combination of techniques, including scar revision, skin excision, and inferior pole remodeling, has been described. Elsahy has described the excision of just the redundant skin along the inverted T scar, but this pulls the NAC downward rather than actually lowering it. 9 Millard et al described two cases in which a combination of techniques was used, including shortening the distance from the nipple to the inframammary line, repositioning of the areola–nipple component, and placement of an implant for the projection of the breast behind the nipple. 10

Grade 3 or moderate to severe nipple elevation (to which grading the case described belonged) not correctable by skin excision and inferior pole remodeling may require upper pole expansion insertion of an implant or lipoaugmentation. If the skin envelope cannot accommodate additional upper pole volume, tissue expansion may be required. Colwell et al have described infraclavicular tissue expansion. It is a staged procedure that requires new incisions. 7

Direct nipple movement through a free graft or reciprocal transposition flap is preferred when there is a short sternal notch to nipple distance combined with a reasonable nipple to IMF distance (5–7 cm). 10 11 12 13 14 15 Z-plasty transposition and V-Y repositioning too have been described to relocate the NAC to a lower position following breast reconstruction. 13

Although the NAC is repositioned lower with all these techniques, there is unfortunately some inevitable scarring. The technique described by the authors avoids new scars and serves the dual purpose of repositioning the NAC to a more acceptable position as well as addressing bottoming out. The only limitation of this technique is the slight but inadvertent shortening of the ideal distance between the NAC and the clavicle, but practically, as our results have shown, it appears to be a good trade-off for fresh scarring in that area as in other techniques.
